# Determinants of the psychomotor development delay in children aged 12 to 59 months infected with HIV in Yaounde, Cameroon

**DOI:** 10.11604/pamj.2022.42.114.33195

**Published:** 2022-06-10

**Authors:** Ginette Claude Mireille Kalla, Ursule Larissa Temgoua Dongmo, Jules Clément Nguedia Assob, Nelly Kamgaing Noubi, Francois-Xavier Mbopi-Keou, Francisca Monebenimp

**Affiliations:** 1 Faculty of Medicine and Biomedical Science, University of Yaounde I, Yaounde, Cameroon,; 2 Faculty of Health Science, University of Buea, Buea, Cameroon,; 3 The Institute for the Development of Africa (The-IDA), Yaounde, Cameroon,; 4 Information Communication Technology University (ICT-U), Yaounde, Cameroon

**Keywords:** Psychomotor delay, children, HIV, Yaounde, Cameroon

## Abstract

**Introduction:**

children infected with HIV are at increased risk of impaired neurodevelopmental, due to several environmental factors.

**Methods:**

we conducted a cross-sectional analytical study on HIV-infected children aged 12 to 59 months, followed up in five hospitals in Yaounde, Cameroon. Sociodemographic, clinical, and biological variables as well as the antecedents were collected. Data analysis was performed using Statistical Package for the Social Sciences (SPSS) version 25 software. The Denver test was used to assess the psychomotor development of these children. Global psychomotor delay, defined as a global development quotient of less than 70 with an alteration in at least two of the four domains of the test, was retained as the primary endpoint. The significance threshold was set at 5%.

**Results:**

one hundred and eighty-one children were included in the study. The sex ratio was 0.6. The age range 48-59 months was the most represented. None of these children had a known chronic pathology other than HIV infection. The proportion of global psychomotor delay was 11.04%, with language (16%) and fine motor skills (16%) being the most affected domains of psychomotor development. The independent factors significantly associated with global psychomotor delay were birth weight below 2500 grams (OR= 17.61 [1.76-181.39], p= 0.022), growth retardation (OR= 17.64 [1.63-190.24], p= 0.018) and elevated viral load (OR= 22.75 [2.78-186.02], p= 0.004).

**Conclusion:**

psychomotor delay affects about one out of ten children living with HIV. Its occurrence is linked to various factors that must be taken into account in the development of public health policies in connection with the management of HIV infection in children.

## Introduction

HIV infection remains a major global public health problem, affecting all age groups, including children. In 2021, the Joint United Nations Program on HIV/AIDS (UNAIDS) reported that 37.7 million people are estimated to be living with HIV, including 1.7 million children under the age of 15 [1]. Due to it's neurotropism, the virus poses a serious threat to the neurological development of affected children. Indeed, it has been found in children infected with HIV that change in the structure of their brain leads to progressive cell destruction, which is all the more serious as it affects structures still under development which should help the acquisition of psychomotor and cognitive skills. The result is a slowing down or even a delay in development, of varying severity [2]. In developing countries, HIV-infected children are also exposed to multiple other risk factors for neurodevelopmental delay such as, prematurity, low birth weight, stunting, wasting, low socioeconomic status, parental or caregiver unemployment, lower education level, and many others preventing them from reaching their full neurological potential [3]. In 2018, a systematic review by McHenry *et al*. of 46 studies from countries on almost every continent found higher rates of severe cognitive delay in HIV-infected children compared to their non-HIV-infected peers [4]. In 2019, a South African study by Wedderburn *et al*. on children born to HIV-positive but uninfected mothers found that they were significantly delayed in language acquisition compared to children born to HIV-negative mothers [5]. In Cameroon, a study by Debaudrap *et al*. in 2018 showed that perinatal HIV-infected children had poorer cognitive development and increased behavioral difficulties compared to those who were not [6]. Increasing access to antiretroviral treatment (ART) for both mothers and infected children has improved the prognosis of these children. In Kenya in 2018, Gomez *et al*. reported improvements not only in motor skills, but also in immunological and nutritional status, visible as early as the sixth month of ART [7]. However, the effects of treatment are not always optimal. While ART helps to prevent the worsening of developmental delays in children, it does not always reverse disorders already present at the time of initiation [8]. Based on this observation, in order to preserve the growth and development of children infected with HIV, diagnosis and early initiation of ART is necessary, but the early identification of modifiable environmental factors that may have a negative impact on the psychomotor development of these children is essential. This is the reason why, we decided to carry out this study, with the aim of identify the determinants of psychomotor delay in HIV-infected children aged between 12 and 59 months in Yaounde.

**Objective:** identify the determinants of psychomotor delay in HIV-infected children aged 12 to 59 months.

## Methods

**Study design:** we conducted a cross-sectional and analytic study with prospective data collection from February to May 2021 (4 months) in five health facilities in Yaounde. The approved treatment centers (ATC) of the Yaounde University Teaching Hospital (YUTH), the Yaounde Gynaeco-Obstetric and Paediatric Hospital (YGOPH), and the Mother and Child Center of the Chantal Biya Foundation (MCC-FCB), the treatment units (TU) of the District Hospitals of Efoulan and Cité-Verte.

**Study participants:** the sample size was obtained using the formula for cross-sectional studies. Based on the proportion of 12% of global psychomotor retardation found in Amsterdam in 2013 by van Arnhem *et al*. in a population of HIV-infected children [9], we obtained a minimum sample size of 163 participants. Adding a non-response percentage of 10% gave us a minimum sample size of 180 children. Children aged 12 to 59 months, infected with HIV, followed for at least 6 months in one of the five hospitals retained for the study and for whom at least one parent/guardian had signed the inform consent form were include. We excluded children with a diagnosis of cerebral palsy, spina bifida, or with an episode of acute illness in the month prior to the study. They were enrolled consecutively.

**Procedures:** after having obtained the approval of the Ethics and Research Committee of the Faculty of Medicine and Biomedical Sciences of the University of Yaounde I and the research authorization of the Director of the five health facilities retained for the study, the children fulfilling our inclusion criteria were selected during the consultation. During this first contact, we explained the aims and the course of the study to the parents and carers we met. An appointment was then made for a later date with the consenting parents/guardians either at the hospital or at the parents' home, in order to fill our questionnaire and to carry out the Denver test under optimal conditions. The day of the appointment, we began by the administration of the questionnaire to the parent or guardian, followed by a physical examination of the child before the Denver test was administered.

**The data collected were:** i) Socio-demographic data of the child and the mother: age and sex of the child, whether the child is an orphan, mother´s age, mother's educational level, mother's occupation, mother's marital status; ii) antecedents: term of birth, birth weight, notion of resuscitation at birth, hospitalisation and duration of hospitalisation at birth, known chronic pathology, number of hospitalisations since birth, age at diagnosis of HIV infection, age of initiation of antiretroviral therapy (ARV) treatment, change in treatment line, compliance with ARV treatment, type of breastfeeding received, age of weaning, number of children under 5 years of age in the sibling group, number of people living in the house; iii) clinical data: head circumference, weight, height, weight-for-height index, height-for-age index, who stage of disease. Biological data: latest viral load. The Denver developmental screening test (DDST) was chosen for this study because, although it was developed in 1967, it is still very much in use today since it is easy to use and understand, accessible and does not pose any cultural adaptation problems [10]. However, it is important to note that this test has some limitations, in that it is not a definitive diagnostic tool, but rather a mean of targeting children requiring closer follow-up. At the end of the assessment, for each domain of the test, two values were determined: i) the developmental age (DA): the age at which the child achieved at least three consecutive skills; ii) the partial developmental quotient (PDQ): calculated as a ratio of the child's developmental age to chronological age, multiplied by 100. Then, a global development quotient (GDQ) was calculated by averaging the obtained PDQs. Psychomotor development was considered to be delayed in any child with a GDQ of less than 70, with impairment in at least two test domains.

**Statistical analysis:** data were entered into census and survey processing system CSPRO) version 7.3 and analyzed using SPSS version 25.0 software. The qualitative variables were expressed as numbers and percentages, while the quantitative variables were expressed as means (or medians), standard deviations (or interquartile ranges) and extremes. For comparison of proportions, chi-square and Fischer tests were used. The strength of association was estimated by odds ratio and the 95% confidence interval. In order to exclude the effect of confounding factors, multivariable analysis was performed using the logistic regression model, including all variables with a p-value less than 0.05. The p-value <0.05 was considered to be statistically significant.

**Ethical considerations:** all the authorizations were obtained. The informed consent form was signed by each parent or guardian of each child included in our study. Confidentiality and anonymity were strictly observed. All children with abnormalities in one or more domains of psychomotor development were referred to a neuropediatrician for appropriate follow-up.

## Results

From February to May 2021, 181 patients meeting our inclusion criteria were selected for the study. Females were the most represented, 109 (60.2%) with a sex ratio of 0.6. Ages ranged from 12 to 59 months with an average of 39.4 ±15.16 months. The most represented age group was [48-59] months (82; 45.3%). The majority, 159 (87.8%) were not orphans, and 75 (41.7%) lived in the same household as both parents. As for the mothers, the age group most represented was [25-35] years (116; 68.6%). The majority of them, 137 (81%), had at least a secondary education. Housewives and professionals were predominant, 68 (40.2%). Single mothers (127; 75.1%) were the most represented (Table 1).

**Table 1:** sociodemographic characteristics of children and mothers

Variables	Frequency (n)	Percentage (%)
**Children (n=181)**		
**Gender**		
Male	72	39.8
Female	109	60.2
**Age (months)**		
[12-24]	34	18.8
[24-36]	37	20.4
[36-48]	28	15.5
[48-59]	82	45.3
**Orphan**		
Yes	22	12.2
No	159	87.8
**Person living with the child**		
Father	10	5,5
Mother	67	37
Both	75	41,7
Tutor	28	15.6
**Mothers (n=169)**		
**Mother's age (years)**		
[18-25]	16	9.5
[25-35]	116	68
≥ 35	37	21,9
**Educational level of the mother**		
None/primary	32	19
Secondary and above	137	81
**Mother's occupation**		
Household	68	40,2
Pupil/student	12	7,2
Civil servant	21	12.4
Liberal profession	68	40.2
**Marital status of the mother**		
Single	127	75.1
Married	42	24.9

**Personal and family antecedents of the study population:** the majority, 167 (92.3%) children were born at term and 22 (71.7%) had a birth weight of [2500-3500] grams. No resuscitation was performed at birth for 98.9% of them. During the neonatal period, 13 (7.2%) of these children were hospitalized, and the most frequent reason was neonatal jaundice (05; 38.5%) followed by neonatal infection (04; 30.8%). None of the study participants had a known chronic condition other than HIV infection, and 66 (36.5%) of them had never been hospitalized since birth. For those who had been hospitalized at least once after the neonatal period, severe malaria (39; 33.9%) was the most reported reason. The 2-9 month age group was the most represented at the time of diagnosis of HIV infection (72; 39.8%) and initiation of ART (71; 39.3%). Adherence was achieved in 155 (85.6%) of the participants, none of whom had experienced any change in ART. The immunization status of (161) 89% of the participants was not up to date for their age. During the first six months of life, 110 (60.8%) of these children were exclusively breastfed. Fifty-six were weaned before 6 months of age (31.5%) (Table 2). At the family level, 101 (55.8%) of these children were at least the third sibling, and in 77.9% of cases, this sibling had only one child under the age of 5. The households in which these children lived were 91.7% composed of at least four people. For 176 (97.2%) of these families, there was no other child infected with HIV (Table 3).

**Table 2:** personnal antecedents of the study population

Variables	Frequency (n=181)	Percentage (%)
**Born at term**		
Yes	167	92.3
No	14	7.7
**Birth weight (in grams)**		
< 2500	22	12,2
[2500-3500]	129	71.7
≥ 3500	29	16.1
**Ressuscitation at birth**		
Yes	2	1.1
No	179	98.9
**Neonatal hospitalization**		
Yes	13	7.2
No	168	92.8
**Known chronic other pathology**		
Yes	0	0
No	181	100
**Number of hospitalizations since birth**		
0	66	36.5
1	77	42.5
≥ 2	38	21
**Reason for each hospitalization**		
Respiratory infection	33	28,6
Digestive infection	26	22.6
Neurological infection	1	0.8
Severe malaria	39	33.9
Malnutrition	16	13.9
**Age at diagnosis of HIV infection (in months)**		
[2-9]	72	39.8
[9-18]	68	37.6
≥ 18	41	22.6
**Compliance with ART**		
Yes	155	85.6
No	26	14.4
**Changes in the ART line**		
Yes	0	0
No	181	100
**Up-to-date vaccination**		
Yes	20	11
No	161	89
**Feeding pattern for the first six months**		
Exclusive feeding	110	60,8
Formula feeding	21	11.6
Mixed feeding	50	27.6
**Age at weaning (in months)**		
< 4	21	11,6
[4-6]	36	19.9
≥ 6	124	68,5

ART : antiretroviral treatment

**Table 3:** family past history of the study population

Variables	Frequency (n=181)	Percentage (%)
**Sibling rank**		
1	29	16
2	51	28.2
≥ 3	101	55.8
**Children under 5 in sibling group**		
1	141	77.9
2	40	22.1
**Number of people living in the household**		
2	5	2.8
3	10	5.5
≥4	166	91.7
**Other infected child in sibling group**		
Yes	5	2.8
No	176	97.2

**Clinical and paraclinical characteristics:** head circumference values were within normal limits in 178 (98.3%) participants. Acute malnutrition was found in 7 (3.9%) of these children, and 37 (20.4%) were stunted. HIV infection was WHO stage 1 in 144 (79.6%) of the participants, and the most recent viral load in 149 (82.3%) of these was less than 1000 copies/ml (Table 4).

**Table 4:** clinical and paraclinical characteristics of the study population

Variables	Frequency (n=181)	Percentage (%)
**Head circunference for age (Z-score)**		
< -2	3	1.7
[-2 , +2]	178	98.3
> +2	0	0
**Weight for height index (Z-score)**		
< -2	7	3.9
[-2;+2]	174	96.1
> +2	0	0
**Height for age index (Z-score)**		
< -2	37	20.4
[-2,+2]	143	79
> +2	1	0.6
**WHO stage of the disease**		
Stage 1	144	79.6
Stage 2	0	0
Stage 3	0	0
Stage 4	37	20.4
**Latest viral load (in copies/ml)**		
< 1000	149	82.3
≥ 1000	32	17.7

WHO: World Health Organisation

**Figure 1:**
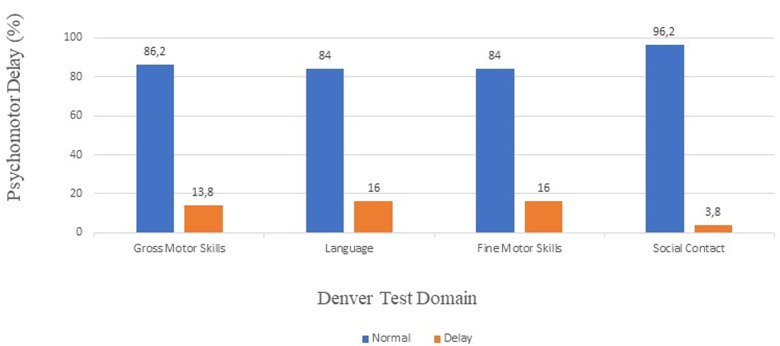
psychomotor assessment of the study population

**Psychomotor assessment of the study population:** of the 181 children included in the study, 20 had global psychomotor delay, representing 11.04%. Language (29; 16%) and fine motor skills (29; 16%) were the domains in which the most delay was recorded (Figure 1).

**Factors associated with the psychomotor development delay:** age between [12-24] months (OR=3.6 [1.33-9.7], p=0.014), living with a guardian (OR=3.53 [1.26-9.88], p=0.02), mothers with less than secondary education (OR=5.52 [1.98-15.39], p=0.002), birth weight of less than 2500 grams (OR=3.83 [1.29-11.35], p= 0.021), children with at least two previous hospitalisations (OR=13.22 [4.62-37.79], p=0.000), non-compliance to ART (OR=5.25 [1.8-14.5], p=0.03), weaning between 4-6 months (OR=4.03 [1.52-10.66], p=0.006), living in a house with fewer than 4 people (OR=5 [1.51-16.56], p=0.015), acute malnutrition (OR=6.88 [1.42-33.36], p=0.031), stunting (OR=39.66 [10.66-147.56], p=0.000), WHO stage 4 disease (OR=28 [8.5-92.18], p= 0.000) and high viral load (OR=25,23 [8.15-78.13], p=0.000) were significantly associated with global psychomotor delay. However, living with both parents (OR=0.21 [0.6-0.75], p=0.014) and exclusive breastfeeding (0.37 [0.14-0.98], p=0.052) were protective factors against the occurrence of global psychomotor delay.

**Multivariate analysis:** the independent factors significantly associated with global psychomotor delay were: birth weight less than 2500 grams (adjusted OR=17.61 [1.76-181.39], p=0.022), growth retardation (adjusted OR= 17.64 [1.63-190.24], p=0.018), and high viral load (adjusted OR= 22.75 [2.78-186.02], p=0.004).

## Discussion

Among the 181 HIV-infected children in our series, we found a proportion of global psychomotor delay of 11.04%. Our result is similar to the 12% found in Amsterdam in 2013 by Van Arnhem *et al*. [9]. In South Africa, Lentoor *et al*. found a higher rate of cognitive delay in HIV-infected school-aged children compare to our result 13.8% [11]. This difference may be explained by the fact that these children were from one of the poorest provinces in South Africa with a high level of underdevelopment and underemployment leading to poverty who is a factor that negatively influences neurocognitive performance in children [12]. However, in a study conducted in Harare in 2011, Kandawasvika *et al*. found a global psychomotor delay of 10.34% in a population of 3-month-old HIV-infected children were at high risk of impaired neurodevelopment [13]. In our study, each domain taken individually, language and fine motor skills were those in which the most delay had been recorded Sherr *et al*. in a systematic review in 2014 confirm that some domains measured seem to be more affected than others, with mixed evidence on language and executive functioning [14]. Knox *et al*., in a study in South Africa in 2018, found that HIV positive children were more likely to have cognitive delay (OR=2.2, 95% CI=1.2-3.9) and language delay (OR=4.3, 95% CI=2.2-8.4) [15]. A significant association was found between global psychomotor delay and age between 12 and 24 months. This could be explained by the fact that children in this age group are still in the first 1000 days of life, which is a particularly sensitive period for their development during which rapid growth of brain structures is observed. Even the slightest disruption of this process can have long-term consequences for the functional abilities of the brain [16]. Our study found a higher risk of global psychomotor delay in children living with a guardian and in those whose mothers had less than secondary school education. Similar findings were made in 2019 by Lentoor *et al*. in a population of South African school-age children [11]. Boyede *et al*. in 2013, in Nigeria found a threefold higher risk of poor cognitive development in children whose mothers had a primary education or were illiterate [17].

To corroborate this, a study by Pedrini *et al*. in Mozambique in 2015 found that illiterate caregivers were 2.6 times more likely to have a child with delayed psychomotor development compared to caregivers who could read and write [18]. Indeed, neurodevelopmental delays in HIV-infected children are not only attributable to the infection itself, but also to social factors and the environment in which the child lives [17,19]. In our study, the risk of psychomotor delay was higher in children living in a household with less than 4 inhabitants. This could be explained by the fact that cognitive stimulation and play would be less frequent the smaller the number of inhabitants in the household [17]. A significant association has been found between low birth weight and global psychomotor delay. This is not unique to children living with HIV, as cognitive delay is generally one of the most common sequelae in very low birth weight preterm infants [13]. In 2015, Linsell *et al*. highlighted low birth weight as a predictor of global cognitive impairment in children under 5 years of age [20]. Children in our sample who had been hospitalised at least twice were at greater risk of developing global psychomotor delay. Due to the late diagnosis of HIV infection in children in sub-Saharan Africa, they have a higher susceptibility to develop various infectious comorbidities and organ dysfunctions, due to prolonged immunodeficiency during childhood [18,21]. A high risk of psychomotor delay was found in non-adherent children to antiretroviral therapy (ART). Nowadays, it is known that the brains of infected individuals may be the site of low viral replication, leading to a local inflammatory cascade involving cytokines and chemokines, which can damage neurons and thus have a deleterious effect on child neurological development. The use of ARTs able to penetrate the blood-brain barrier may reduce the incidence of these complications [22]. Stauch reported that there is evidence of an association between neurocognitive ability and adherence to ART [23].

We also found that advanced disease stage was significantly associated with the occurrence of global psychomotor delay. This finding is consistent with a 2018 study by Gomez *et al*. in Kenya, which found lower psychomotor performance in children under 5 years old with advanced infection [7]. Other studies have found more cognitive impairment, memory impairment and learning difficulties in similar populations [24-26]. Similarly, poor nutritional status and high viral load were significantly associated with the occurrence of global psychomotor delay in our study population. Progressive stunting is considered one of the most common abnormalities in children with perinatal HIV infection [27]. Poor nutrition has been associated with weakened immunity and accelerated disease progression in children living with HIV [28]. In addition, people living with HIV are generally vulnerable to food insecurity due to reduced economic capacity, and food insecurity has been associated with reduced access to care and poorer clinical outcomes for people living with HIV (PLHIV) [29]. Reduced immunity will lead to increased and rapid replication of the virus and the production of high levels of certain immunological markers, resulting in behavioural disturbances, as described by Ruisenor-Escudero *et al*. in 2015 in a population of Ugandan school children [30].

In addition to the risk factors mentioned above, our study also highlighted some protective factors for harmonious psychomotor development. Among these, living with both parents was found. A similar conclusion has been made by Nanthamongkolchai in 2015 in Thailand showing that children reared by a grandparent had 2.9 times higher chance of delayed development than those who were reared by their parents [31]. Another protective factor was exclusive breastfeeding. Indeed, the developing brain is vulnerable to the effects of sub-optimal maternal nutrition, as the nutrition provided by the mother before birth through transplacental transfer and after birth through exclusive breastfeeding and other enteral foods, promotes rapid fetal and neonatal brain development [32]. Exclusive breastfeeding is recommended for women living with HIV on ART, especially as undernutrition, diarrhoea and pneumonia are common causes of infant mortality [33]. It has also been associated with fewer hospital admissions for infants exposed to HIV in the first year of life [34].

## Conclusion

One HIV-infected child out on 10 have a global psychomotor delay. The independent factors significantly associated with global psychomotor delay were: birth weight less than 2500 grams, growth retardation and high viral load. This study shows that there are many factors that must be taken into account to prevent psychomotor delay in children infected with HIV in our context.

### What is known about this topic

- Children infected with HIV are at increased risk of impaired neurodevelopment, due to several environmental factors.

### What this study adds

- One HIV-infected child out on 10 have a global psychomotor delay;
- The independent factors significantly associated with global psychomotor delay were: birth weight less than 2500 grams, growth retardation and high viral load;
- This study shows that there are many factors that must be taken into account to prevent psychomotor delay in children infected with HIV in our context;

## References

[1] United Nations Programme on HIV/AIDS (2020). Global HIV and AIDS statistics; fact sheet.

[2] Yadav SK, Gupta RK, Garg RK, Venkatesh V, Gupta PK, Singh AK (2017). Altered structural brain changes and neurocognitive performance in pediatric HIV. Neuroimage Clin.

[3] Blokhuis C, Kootstra NA, Caan MW, Pajkrt D (2016). Neuro developmental delay in pediatric HIV/AIDS: current perspectives. Neurobehavioral HIV Medicine.

[4] McHenry MS, McAteer CI, Oyungu E, McDonald BC, Bosma CB, Mpofu PB (2018). Neuro development in young children born to HIV-infected mothers: a meta-analysis. Pediatrics.

[5] Wedderburn CJ, Yeung S, Rehman AM, Stadler JAM, Nhapi RT, Barnett W (2019). Neurodevelopment of HIV-exposed uninfected children in South Africa: outcomes from an observational birth cohort study. Lancet Child Adolesc Health.

[6] Debeaudrap P, Bodeau-Livinec F, Pasquier E, Germanaud D, Ndiang ST, Nlend AN (2018). Neurodevelopmental outcomes in HIV-infected and uninfected African children. AIDS.

[7] Gomez LA, Crowell CS, Njuguna I, Cranmer LM, Wamalwa D, Chebet D (2018). Improved neurodevelopment after initiation of antiretroviral therapy in human immunodeficiency virus-infected children. Pediatr Infect Dis J.

[8] Whitehead N, Potterton J, Coovadia A (2014). The neurodevelopment of HIV-infected infants on HAART compared to HIV-exposed but uninfected infants. AIDS Care.

[9] Van Arnhem LA, Bunders MJ, Scherpbier HJ, Majoie CBLM, Reneman L, Frinking O (2013). Neurologic abnormalities in HIV-1 infected children in the era of combination antiretroviral therapy. PLoS One.

[10] Tardieu M (1992). Psychomotor development in children. Elements of estimation. Rev Prat.

[11] Lentoor AG (2019). The association of home environment and caregiver factors with neurocognitive function in pre-school-and school-aged perinatally acquired HIV-positive children on cART in South Africa. Front Pediatr.

[12] Walker SP, Wachs TD, Grantham-McGregor S, Black MM, Nelson CA, Huffman SL (2011). Inequality in early childhood: risk and protective factors for early child development. Lancet.

[13] Kandawasvika GQ, Ogundipe E, Gumbo FZ, Kurewa EN, Mapingure MP, Stray-Pedersen B (2011). Neurodevelopmental impairment among infants born to mothers infected with human immunodeficiency virus and uninfected mothers from three peri-urban primary care clinics in Harare, Zimbabwe: neurodevelopmental impairment in HIV-exposed infants. Dev Med Child Neurol.

[14] Sherr L, Croome N, Parra Castaneda K, Bradshaw K, Herrero Romero R (2014). Developmental challenges in HIV infected children-an updated systematic review. Children and Youth Services Review.

[15] Knox J, Arpadi SM, Kauchali S, Craib M, Kvalsvig JD, Taylor M (2018). Screening for developmental disabilities in HIV positive and HIV negative children in South Africa: results from the Asenze study. PLoS One.

[16] Darling JC, Bamidis PD, Burberry J, Rudolf MCJ (2020). The First thousand days: early, integrated and evidence-based approaches to improving child health: coming to a population near you?. Arch Dis Child.

[17] Boyede G, Lesi FE, Ezeaka C, Umeh CS (2013). Impact of sociodemographic factors on cognitive function in school-aged HIV-infected Nigerian children. HIV AIDS (Auckl).

[18] Pedrini M, Moraleda C, Macete E, Gondo K, Brabin BJ, Menéndez C (2015). Clinical, nutritional and immunological characteristics of HIV-infected children in an area of high HIV prevalence. J Trop Pediatr.

[19] Miguel PM, Pereira LO, Silveira PP, Meaney MJ (2019). Early environmental influences on the development of children´s brain structure and function. Dev Med Child Neurol.

[20] Linsell L, Malouf R, Morris J, Kurinczuk JJ, Marlow N (2015). Prognostic factors for poor cognitive development in children born very preterm or with very low birth weight: a systematic review. JAMA Pediatr.

[21] Lowenthal ED, Bakeera-Kitaka S, Marukutira T, Chapman J, Goldrath K, Ferrand RA (2014). Perinatally acquired HIV infection in adolescents from sub-Saharan Africa: a review of emerging challenges. Lancet Infect Dis.

[22] Borrajo A, Spuch C, Penedo MA, Olivares JM, Agís-Balboa RC (2021). Important role of microglia in HIV-1 associated neurocognitive disorders and the molecular pathways implicated in its pathogenesis. Ann Med.

[23] Peterson PK, Toborek M (2014). Neuroinflammation and neurodegeneration. Springer New York.

[24] Crowell CS, Malee KM, Yogev R, Muller WJ (2014). Neurologic disease in HIV-infected children and the impact of combination antiretroviral therapy: HIV-associated neurologic disease in children. Rev Med Virol.

[25] Smith R, Chernoff M, Williams PL, Malee KM, Sirois PA, Kammerer B (2012). Impact of HIV severity on cognitive and adaptive functioning during childhood and adolescence. Pediatr Infect Dis J.

[26] Nichols SL, Chernoff MC, Malee K, Sirois PA, Williams PL, Figueroa V (2016). Learning and memory in children and adolescents with perinatal HIV infection and perinatal HIV exposure. Pediatr Infect Dis J.

[27] Yasuoka J, Yi S, Okawa S, Tuot S, Murayama M, Huot C (2020). Nutritional status and dietary diversity of school-age children living with HIV: a cross-sectional study in Phnom Penh, Cambodia. BMC Public Health.

[28] Chiabi A, Lebela J, Kobela M, Mbuagbaw L, Obama MT, Ekoe T (2012). The frequency and magnitude of growth failure in a group of HIV-infected children in Cameroon. Pan Afr Med J.

[29] Anema A, Vogenthaler N, Frongillo EA, Kadiyala S, Weiser SD (2009). Food insecurity and HIV/AIDS: current knowledge, gaps, and research priorities. Curr HIV/AIDS Rep.

[30] Ruiseñor-Escudero H, Familiar I, Nakasujja N, Bangirana P, Opoka R, Giordani B (2015). Immunological correlates of behavioral problems in school-aged children living with HIV in Kayunga, Uganda. Glob Ment Health (Camb).

[31] Nanthamongkolchai S, Munsawaengsub C, Nanthamongkolchai C (2009). Influence of child rearing by grandparent on the development of children aged six to twelve years. J Med Assoc Thai.

[32] Cusick SE, Georgieff MK (2016). The role of nutrition in brain development: the golden opportunity of the “First 1000 Days”. J Pediatr.

[33] World Health Organization, United Nations Children´s Fund (2016). Guideline: updates on HIV and infant feeding.

[34] Asbjörnsdóttir KH, Slyker JA, Maleche-Obimbo E, Wamalwa D, Otieno P, Gichuhi CM (2016). Breastfeeding is associated with decreased risk of hospitalization among HIV-exposed, uninfected Kenyan infants. J Hum Lact.

